# (2*R*,4*R*)-4-(2-Eth­oxy-2-oxoeth­yl)-2,6,6-trimeth­yl–2-oxo-1,3,6,2λ^5^-dioxaza­phospho­can-6-ium iodide

**DOI:** 10.1107/S1600536812018466

**Published:** 2012-05-02

**Authors:** Bradford W. Fulfer, Frank R. Fronczek, Steven F. Watkins

**Affiliations:** a Department of Chemistry, Louisiana State University, Baton Rouge, LA 70803-1804, USA

## Abstract

The title compound, C_11_H_23_NO_5_P^+^·I^−^, consists of an eight-membered cationic heterocyclic ring in a boat–chair conformation. The ring features a tetra­alkyl­ammonium N and a methyl­phospho­nate P atom. A –CH_2_(CO)OC_2_H_5_ ester side chain at the C adjacent to oxygen produces two chiral centers at that substituted C atom and the P atom, both of which were determined to have absolute *R*,*R* configurations. A previously determined racemic bromide analog has exactly the same ring but with a –C_15_H_31_ side chain. In that structure, both chiral centers show the same relative *R*/*S*,*R*/*S* configurations, but the ring in the bromide analog is in a boat conformation.

## Related literature

For *MM2* energy minimization, see Cambridgesoft (2010 ▶). For a description of the Cambridge Structural Database, see: Allen (2002 ▶). For the absolute configuration from Bijvoet pair analysis, see: Hooft *et al.* (2008 ▶). For the synthesis, see: Kumaravel *et al.* (1994 ▶); Hubieki *et al.* (1996 ▶). For a related structure, see: Kumaravel *et al.* (1995 ▶).
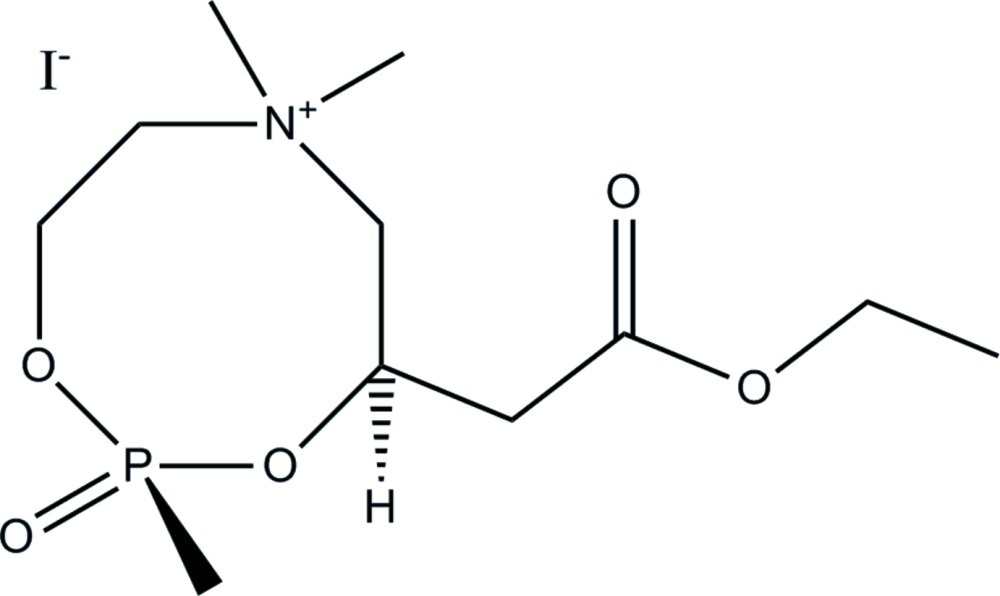


## Experimental

### 

#### Crystal data

C_11_H_23_NO_5_P^+^·I^−^
*M* *_r_* = 407.17
Orthorhombic, 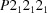

*a* = 7.4882 (2) Å
*b* = 11.7438 (2) Å
*c* = 18.0235 (4) Å
*V* = 1584.99 (6) Å^3^
*Z* = 4
Mo *K*α radiation
μ = 2.14 mm^−1^
*T* = 90 K
0.28 × 0.25 × 0.15 mm

#### Data collection

Nonius KappaCCD diffractometer
Absorption correction: multi-scan (*SCALEPACK*; Otwinowski & Minor, 1997 ▶) *T*_min_ = 0.591, *T*_max_ = 0.740
26155 measured reflections
6304 independent reflections
6235 reflections with *I* > 2σ(*I*)
*R*_int_ = 0.042

#### Refinement

*R*[*F* ^2^ > 2σ(*F* ^2^)] = 0.016
*wR*(*F* ^2^) = 0.039
*S* = 1.05
6304 reflections
177 parameters
H-atom parameters constrained
Δρ_max_ = 0.39 e Å^−3^
Δρ_min_ = −0.66 e Å^−3^
Absolute structure: Flack (1983 ▶), 2741 Friedel pairs
Flack parameter: 0.006 (7)

### 

Data collection: *COLLECT* (Bruker 2004 ▶); cell refinement: *SCALEPACK* (Otwinowski & Minor, 1997 ▶); data reduction: *DENZO* (Otwinowski & Minor, 1997 ▶), *SCALEPACK* and *SORTAV* (Blessing, 1987 ▶, 1989 ▶); program(s) used to solve structure: *SHELXS97* (Sheldrick, 2008 ▶); program(s) used to refine structure: *SHELXL97* (Sheldrick, 2008 ▶); molecular graphics: *ORTEP-3 for Windows* (Farrugia, 1997 ▶); software used to prepare material for publication: *WinGX* (Farrugia, 1999 ▶).

## Supplementary Material

Crystal structure: contains datablock(s) global, I. DOI: 10.1107/S1600536812018466/pv2538sup1.cif

Structure factors: contains datablock(s) I. DOI: 10.1107/S1600536812018466/pv2538Isup2.hkl

Supplementary material file. DOI: 10.1107/S1600536812018466/pv2538Isup3.cml

Additional supplementary materials:  crystallographic information; 3D view; checkCIF report

## Figures and Tables

**Table 1:** Selected torsion angles (°)

O2—P1—O1—C3	−68.45 (9)
P1—O1—C3—C4	77.51 (10)
O1—C3—C4—N1	−111.53 (11)
C3—C4—N1—C1	52.59 (13)
C4—N1—C1—C2	57.38 (13)
N1—C1—C2—O2	−64.93 (14)
C1—C2—O2—P1	−49.45 (14)
C2—O2—P1—O1	103.91 (10)

**Fig. 1.**
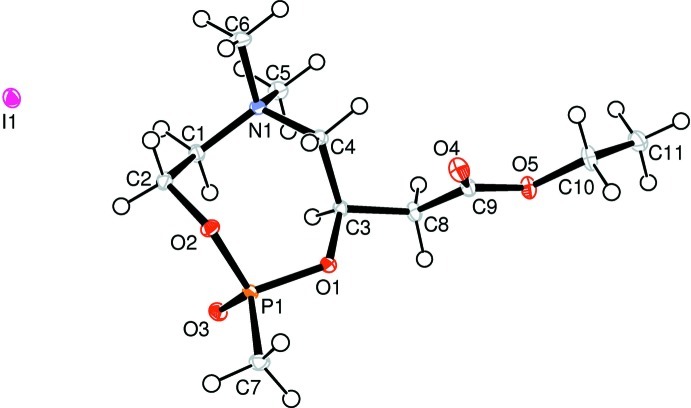
View of (I) (50% probability displacement ellipsoids)

## supplementary crystallographic information

### Comment

The title compound (I) is an analog of 2,6,6-trimethyl-2-oxo-1,3-dioxa- 6-azonia-2-phosphocyclooctane bromide (compound II, CSD code YEVZUU, Allen, 2002), originally targeted for use as a reaction-intermediate inhibitor of carnitine acyltransferase (Kumaravel *et al.*, 1994; Kumaravel *et al.*, 1995). Both compounds contain the same 8-membered cationic heterocyclic ring (C_7_H_16_NO_3_P) with two chiral centers at C3 and P1, but with different side chains at C3.

Compound I crystallizes as the *R,R* enantiomer, whereas II crystallizes as a racemate with *R/S,R/S* relative configurations. In I, the ring is in the boat-chair conformation, the lowest energy conformer of paradigmatic cyclooctane (*Chem3DPro*, Cambridgesoft, 2010), but surprisingly the ring in II is in the boat conformation, which in cyclooctane is a higher energy conformer.

### Experimental

Synthesis of this class of compounds has been described (Kumaravel *et al.*, 1994; Hubieki *et al.*, 1996). A suitable single-crystal was kindly supplied by Dr. J. H. Rouden.

### Refinement

All H atoms were placed in calculated positions guided by difference maps. The C—H bond distances were constrained to the range from 0.98 to 1.00 Å, and *U*_iso_= 1.2*U*_eq_ (1.5 for methyl groups), thereafter refined as riding. A torsional parameter was refined for each methyl group.

The absolute configuration was determined by analysis of Bijvoet pairs: the Flack (Flack, 1983) parameter = 0.006 (7), the Hooft (Hooft *et al.*, 2008) parameter = 0.006 (6) and P2(true) = 1.000.

### Crystal data

**Table:**

C_11_H_23_NO_5_P^+^·I^−^	*F*(000) = 816
*M**_r_* = 407.17	*D*_x_ = 1.706 Mg m^−^^3^
Orthorhombic, *P*2_1_2_1_2_1_	Mo *K*α radiation, λ = 0.71073 Å
Hall symbol: P 2ac 2ab	Cell parameters from 3522 reflections
*a* = 7.4882 (2) Å	θ = 2.5–33.7°
*b* = 11.7438 (2) Å	µ = 2.14 mm^−^^1^
*c* = 18.0235 (4) Å	*T* = 90 K
*V* = 1584.99 (6) Å^3^	Prism, colourless
*Z* = 4	0.28 × 0.25 × 0.15 mm

### Data collection

**Table:**

Nonius KappaCCD diffractometer	6304 independent reflections
Radiation source: sealed tube	6235 reflections with *I* > 2σ(*I*)
Graphite monochromator	*R*_int_ = 0.042
Detector resolution: 9 pixels mm^-1^	θ_max_ = 33.7°, θ_min_ = 2.9°
CCD rotation images, thick slices scans	*h* = −11→11
Absorption correction: multi-scan (*SCALEPACK*; Otwinowski & Minor, 1997)	*k* = −18→18
*T*_min_ = 0.591, *T*_max_ = 0.740	*l* = −27→28
26155 measured reflections	

### Refinement

**Table:**

Refinement on *F*^2^	Hydrogen site location: inferred from neighbouring sites
Least-squares matrix: full	H-atom parameters constrained
*R*[*F*^2^ > 2σ(*F*^2^)] = 0.016	*w* = 1/[σ^2^(*F*_o_^2^) + (0.0158*P*)^2^ + 0.6491*P*] where *P* = (*F*_o_^2^ + 2*F*_c_^2^)/3
*wR*(*F*^2^) = 0.039	(Δ/σ)_max_ = 0.001
*S* = 1.05	Δρ_max_ = 0.39 e Å^−^^3^
6304 reflections	Δρ_min_ = −0.66 e Å^−^^3^
177 parameters	Extinction correction: *SHELXL97* (Sheldrick, 2008), Fc^*^=kFc[1+0.001xFc^2^λ^3^/sin(2θ)]^-1/4^
0 restraints	Extinction coefficient: 0.0037 (2)
0 constraints	Absolute structure: Flack (1983), 2741 Friedel pairs
Primary atom site location: structure-invariant direct methods	Flack parameter: 0.006 (7)
Secondary atom site location: difference Fourier map	

### Special details

**Table:**

Geometry. All e.s.d.'s (except the e.s.d. in the dihedral angle between two l.s. planes) are estimated using the full covariance matrix. The cell e.s.d.'s are taken into account individually in the estimation of e.s.d.'s in distances, angles and torsion angles; correlations between e.s.d.'s in cell parameters are only used when they are defined by crystal symmetry. An approximate (isotropic) treatment of cell e.s.d.'s is used for estimating e.s.d.'s involving l.s. planes.
Refinement. Refinement of *F*^2^ against ALL reflections. The weighted *R*-factor *wR* and goodness of fit *S* are based on *F*^2^, conventional *R*-factors *R* are based on *F*, with *F* set to zero for negative *F*^2^. The threshold expression of *F*^2^ > 2σ(*F*^2^) is used only for calculating *R*-factors(gt) *etc*. and is not relevant to the choice of reflections for refinement. *R*-factors based on *F*^2^ are statistically about twice as large as those based on *F*, and *R*- factors based on ALL data will be even larger.

### Fractional atomic coordinates and isotropic or equivalent isotropic displacement parameters (Å^2^)

**Table:**

	*x*	*y*	*z*	*U*_iso_*/*U*_eq_	
I1	0.043921 (11)	0.959574 (7)	0.228805 (4)	0.01449 (2)	
C1	0.36047 (17)	0.73826 (11)	0.14505 (7)	0.0118 (2)	
H1A	0.4184	0.762	0.0982	0.014*	
H1B	0.3399	0.808	0.1747	0.014*	
C2	0.17996 (17)	0.68738 (12)	0.12605 (7)	0.0127 (2)	
H2A	0.122	0.662	0.1726	0.015*	
H2B	0.1042	0.7478	0.1042	0.015*	
C3	0.59764 (16)	0.56554 (10)	0.06758 (7)	0.0098 (2)	
H3	0.6129	0.6486	0.0573	0.012*	
C4	0.53465 (16)	0.55152 (10)	0.14844 (6)	0.01054 (18)	
H4A	0.4272	0.5024	0.149	0.013*	
H4B	0.6293	0.5121	0.1769	0.013*	
C5	0.66050 (17)	0.73081 (11)	0.19789 (7)	0.0135 (2)	
H5A	0.6346	0.7997	0.2266	0.02*	
H5B	0.7092	0.7522	0.1494	0.02*	
H5C	0.7479	0.6842	0.2246	0.02*	
C6	0.41775 (17)	0.63424 (12)	0.26270 (7)	0.0157 (2)	
H6A	0.5067	0.59	0.2903	0.024*	
H6B	0.3084	0.5892	0.2572	0.024*	
H6C	0.3908	0.7045	0.2898	0.024*	
C7	0.17486 (17)	0.50423 (12)	−0.06268 (7)	0.0133 (2)	
H7A	0.0611	0.54	−0.0761	0.02*	
H7B	0.1518	0.431	−0.0385	0.02*	
H7C	0.2462	0.4919	−0.1075	0.02*	
C8	0.77289 (16)	0.50631 (11)	0.05177 (7)	0.0119 (2)	
H8A	0.7942	0.5088	−0.0024	0.014*	
H8B	0.8696	0.5504	0.0758	0.014*	
C9	0.78769 (17)	0.38388 (11)	0.07689 (7)	0.0118 (2)	
C10	1.00454 (18)	0.23653 (11)	0.09371 (9)	0.0171 (2)	
H10A	0.964	0.1762	0.0592	0.021*	
H10B	0.9495	0.223	0.1429	0.021*	
C11	1.2050 (2)	0.23625 (13)	0.09977 (8)	0.0180 (2)	
H11A	1.2573	0.2507	0.0508	0.027*	
H11B	1.2453	0.162	0.118	0.027*	
H11C	1.2429	0.2959	0.1344	0.027*	
N1	0.49050 (13)	0.66341 (9)	0.18714 (6)	0.01052 (17)	
O1	0.46590 (12)	0.52051 (7)	0.01612 (4)	0.01006 (14)	
O2	0.18599 (12)	0.59216 (8)	0.07507 (5)	0.01138 (16)	
O3	0.32941 (13)	0.71123 (8)	−0.02761 (5)	0.01296 (17)	
O4	0.66875 (13)	0.32687 (9)	0.10299 (6)	0.01603 (18)	
O5	0.95505 (14)	0.34845 (8)	0.06562 (5)	0.01394 (16)	
P1	0.29315 (4)	0.59443 (3)	−0.001033 (16)	0.00900 (5)	

### Atomic displacement parameters (Å^2^)

**Table:**

	*U*^11^	*U*^22^	*U*^33^	*U*^12^	*U*^13^	*U*^23^
I1	0.01306 (3)	0.01533 (4)	0.01509 (4)	0.00285 (3)	−0.00355 (3)	−0.00370 (3)
C1	0.0108 (5)	0.0117 (5)	0.0129 (5)	0.0012 (4)	−0.0012 (4)	−0.0008 (4)
C2	0.0098 (5)	0.0163 (6)	0.0121 (5)	0.0020 (4)	0.0004 (4)	−0.0031 (4)
C3	0.0081 (4)	0.0108 (5)	0.0105 (4)	0.0001 (3)	0.0001 (3)	0.0006 (4)
C4	0.0108 (4)	0.0110 (5)	0.0098 (4)	0.0004 (4)	−0.0003 (4)	0.0010 (3)
C5	0.0111 (5)	0.0154 (5)	0.0141 (5)	−0.0038 (4)	−0.0010 (4)	−0.0012 (4)
C6	0.0158 (5)	0.0223 (6)	0.0090 (5)	−0.0027 (4)	0.0016 (4)	0.0004 (4)
C7	0.0119 (5)	0.0172 (5)	0.0109 (5)	−0.0009 (4)	−0.0015 (4)	−0.0015 (4)
C8	0.0077 (4)	0.0122 (5)	0.0159 (5)	0.0005 (4)	0.0013 (4)	0.0026 (4)
C9	0.0107 (5)	0.0121 (5)	0.0125 (5)	0.0016 (4)	−0.0011 (4)	−0.0001 (4)
C10	0.0149 (6)	0.0109 (5)	0.0256 (6)	0.0038 (4)	−0.0005 (5)	0.0033 (4)
C11	0.0147 (6)	0.0216 (6)	0.0179 (6)	0.0048 (5)	−0.0014 (5)	0.0021 (5)
N1	0.0093 (4)	0.0133 (4)	0.0089 (4)	−0.0003 (3)	0.0002 (3)	−0.0006 (3)
O1	0.0089 (3)	0.0120 (4)	0.0093 (3)	0.0011 (3)	−0.0009 (3)	−0.0004 (3)
O2	0.0102 (4)	0.0143 (4)	0.0096 (4)	−0.0010 (3)	0.0013 (3)	−0.0013 (3)
O3	0.0144 (4)	0.0123 (4)	0.0121 (4)	0.0011 (3)	0.0003 (3)	0.0031 (3)
O4	0.0118 (4)	0.0152 (4)	0.0211 (4)	−0.0009 (3)	0.0010 (3)	0.0039 (4)
O5	0.0107 (4)	0.0121 (4)	0.0191 (4)	0.0023 (3)	0.0016 (4)	0.0026 (3)
P1	0.00772 (12)	0.01135 (13)	0.00792 (11)	0.00067 (10)	−0.00033 (10)	0.00100 (10)

### Geometric parameters (Å, º)

**Table:**

C1—N1	1.5153 (16)	C6—H6C	0.98
C1—C2	1.5170 (18)	C7—P1	1.7723 (13)
C1—H1A	0.99	C7—H7A	0.98
C1—H1B	0.99	C7—H7B	0.98
C2—O2	1.4481 (16)	C7—H7C	0.98
C2—H2A	0.99	C8—C9	1.5115 (18)
C2—H2B	0.99	C8—H8A	0.99
C3—O1	1.4536 (15)	C8—H8B	0.99
C3—C8	1.5123 (17)	C9—O4	1.2095 (16)
C3—C4	1.5407 (16)	C9—O5	1.3361 (16)
C3—H3	1	C10—O5	1.4565 (16)
C4—N1	1.5239 (15)	C10—C11	1.505 (2)
C4—H4A	0.99	C10—H10A	0.99
C4—H4B	0.99	C10—H10B	0.99
C5—N1	1.5115 (16)	C11—H11A	0.98
C5—H5A	0.98	C11—H11B	0.98
C5—H5B	0.98	C11—H11C	0.98
C5—H5C	0.98	O1—P1	1.5883 (9)
C6—N1	1.5063 (16)	O2—P1	1.5893 (9)
C6—H6A	0.98	O3—P1	1.4780 (10)
C6—H6B	0.98		
			
N1—C1—C2	117.20 (10)	P1—C7—H7C	109.5
N1—C1—H1A	108	H7A—C7—H7C	109.5
C2—C1—H1A	108	H7B—C7—H7C	109.5
N1—C1—H1B	108	C9—C8—C3	116.40 (10)
C2—C1—H1B	108	C9—C8—H8A	108.2
H1A—C1—H1B	107.2	C3—C8—H8A	108.2
O2—C2—C1	114.81 (10)	C9—C8—H8B	108.2
O2—C2—H2A	108.6	C3—C8—H8B	108.2
C1—C2—H2A	108.6	H8A—C8—H8B	107.3
O2—C2—H2B	108.6	O4—C9—O5	125.27 (12)
C1—C2—H2B	108.6	O4—C9—C8	126.10 (12)
H2A—C2—H2B	107.5	O5—C9—C8	108.63 (11)
O1—C3—C8	107.54 (10)	O5—C10—C11	106.31 (11)
O1—C3—C4	110.91 (9)	O5—C10—H10A	110.5
C8—C3—C4	113.25 (10)	C11—C10—H10A	110.5
O1—C3—H3	108.3	O5—C10—H10B	110.5
C8—C3—H3	108.3	C11—C10—H10B	110.5
C4—C3—H3	108.3	H10A—C10—H10B	108.7
N1—C4—C3	114.03 (9)	C10—C11—H11A	109.5
N1—C4—H4A	108.7	C10—C11—H11B	109.5
C3—C4—H4A	108.7	H11A—C11—H11B	109.5
N1—C4—H4B	108.7	C10—C11—H11C	109.5
C3—C4—H4B	108.7	H11A—C11—H11C	109.5
H4A—C4—H4B	107.6	H11B—C11—H11C	109.5
N1—C5—H5A	109.5	C6—N1—C5	107.93 (9)
N1—C5—H5B	109.5	C6—N1—C1	110.62 (10)
H5A—C5—H5B	109.5	C5—N1—C1	107.55 (10)
N1—C5—H5C	109.5	C6—N1—C4	107.23 (9)
H5A—C5—H5C	109.5	C5—N1—C4	109.11 (9)
H5B—C5—H5C	109.5	C1—N1—C4	114.23 (9)
N1—C6—H6A	109.5	C3—O1—P1	118.56 (7)
N1—C6—H6B	109.5	C2—O2—P1	123.39 (8)
H6A—C6—H6B	109.5	C9—O5—C10	117.83 (11)
N1—C6—H6C	109.5	O3—P1—O1	114.88 (5)
H6A—C6—H6C	109.5	O3—P1—O2	112.83 (5)
H6B—C6—H6C	109.5	O1—P1—O2	103.53 (5)
P1—C7—H7A	109.5	O3—P1—C7	116.32 (6)
P1—C7—H7B	109.5	O1—P1—C7	101.68 (6)
H7A—C7—H7B	109.5	O2—P1—C7	106.18 (6)
			
O2—P1—O1—C3	−68.45 (9)	C2—C1—N1—C6	−63.71 (13)
P1—O1—C3—C4	77.51 (10)	C2—C1—N1—C5	178.65 (10)
O1—C3—C4—N1	−111.53 (11)	C3—C4—N1—C6	175.54 (10)
C3—C4—N1—C1	52.59 (13)	C3—C4—N1—C5	−67.81 (12)
C4—N1—C1—C2	57.38 (13)	C8—C3—O1—P1	−158.16 (8)
N1—C1—C2—O2	−64.93 (14)	O4—C9—O5—C10	−6.70 (19)
C1—C2—O2—P1	−49.45 (14)	C8—C9—O5—C10	173.21 (11)
C2—O2—P1—O1	103.91 (10)	C11—C10—O5—C9	−159.32 (12)
C8—C3—C4—N1	127.45 (11)	C3—O1—P1—O3	55.03 (9)
O1—C3—C8—C9	−73.74 (13)	C3—O1—P1—C7	−178.47 (9)
C4—C3—C8—C9	49.16 (14)	C2—O2—P1—O3	−20.90 (11)
C3—C8—C9—O4	6.12 (19)	C2—O2—P1—C7	−149.44 (10)
C3—C8—C9—O5	−173.79 (11)		
